# Cross-Sectional Clinical Evaluation of Subantral Augmentation Using Nano Graft Composite: Implications for Implant Success

**DOI:** 10.3390/dj14010057

**Published:** 2026-01-15

**Authors:** Olexiy Kosinov, Olesya Manukhina, Kristina Volchykhina, Oleg Mishchenko, Andrii Liutyi, Agne Ramanaviciute, Vilma Ratautaite, Arunas Ramanavicius

**Affiliations:** 1 Department of Dentistry of Postgraduate Education, Zaporizhzhia State Medical and Pharmaceutical University, 26 Marii Prymachenko Blvd., 69035 Zaporizhzhia, Ukraine; kosinov.o.s@zsmu.edu.ua (O.K.); manukhina.o.m@zsmu.edu.ua (O.M.); mischenko.o.m@zsmu.edu.ua (O.M.); 2 Biomedical Research Centre, Sumy State University, 116, Kharkivska Street, 40007 Sumy, Ukraine; andreylutiy@gmail.com; 3 Institute of Atomic Physics and Spectroscopy, University of Latvia, 3 Jelgavas St, LV-1004 Riga, Latvia; 4 Department of Physical Chemistry, Faculty of Chemistry and Geosciences, Institute of Chemistry, Vilnius University, Naugarduko 24, LT-03225 Vilnius, Lithuania; ar2301@cam.ac.uk; 5 NanoTechnas—Centre of Nanotechnology and Material Science, Faculty of Chemistry and Geosciences, Institute of Chemistry, Vilnius University, Naugarduko 24, LT-03225 Vilnius, Lithuania; vilma.ratautaite@ftmc.lt; 6 MB SensoGrafa, Kiparisų 29, Didzioji Riese, LT-14262 Vilnius, Lithuania

**Keywords:** bone composite, subantral augmentation, osteogenesis, bone regeneration, biocompatibility, dental implantation

## Abstract

**Objectives:** This study aims to evaluate the efficacy of hydroxyapatite-tricalcium phosphate (HAP-TCP) as a bone substitute in subantral augmentation for dental implants. Specifically, it investigates the effects of HAP-TCP on bone quality, density, and integration with implants over time. **Methods:** A prospective controlled longitudinal study was conducted on 22 patients (39–75 years of age) undergoing subantral augmentation and dental implantation. A total of 52 sites of augmented bone and 67 sites of native bone were analyzed using computed tomography (CT) to assess bone density in Hounsfield Units (HU), insertion torque measurements, and the Misch classification for bone quality. Augmented and native bone measurements were compared within each patient. **Results:** The augmented bone exhibited an average density of 1132.6 ± 334.9 HU, which is significantly higher (45.9%) than the average density of native bone at 519.3 ± 395.0 HU. Insertion torque values in the HAP-TCP augmented sites averaged 35 N·cm, showing a 71.4% increase compared to adjacent native bone sites (25 N·cm). The study found notable improvements in bone homogeneity and vascularization within the augmented zones. **Conclusion:** HAP-TCP demonstrates significant potential as a reliable and effective synthetic bone substitute for subantral augmentation in dental implants. It yields higher radiodensity and insertion torque than adjacent native bone, while mitigating complications associated with autogenous grafts. These observational findings support the potential clinical use of HAP-TCP for sinus augmentation.

## 1. Introduction

In daily clinical practice, dentists frequently encounter challenges related to alveolar bone loss, which can result from tooth extraction, severe periodontitis, or surgical interventions for tumors [1,2]. Restoring the edentulous distal maxilla with dental implants is a common clinical issue, which is often complicated by insufficient bone height due to crestal resorption and pneumatization of the maxillary sinus [3,4]. Recently, clinicians have developed surgical augmentation techniques that utilize the existing space within the maxillary sinus to restore bone height and create suitable sites for implant placement [5,6].

Autogenous bone is widely regarded as the gold standard for graft materials, and maxillary sinus implants augmented with autogenous bone grafts have demonstrated high implant survival rates in several studies [7,8,9,10,11]. However, the use of autogenous bone grafts carries risks, including donor site morbidity and unpredictable graft resorption [12,13]. As a result, bone substitutes are being widely adopted to reduce surgical complexity and minimize the need for bone harvesting. Most bone substitutes are exclusively osteoconductive. However, their capacity to facilitate graft maturation and provide long-term support for intraosseous implants is crucial for success [14,15].

Hydroxyapatite (HAP) is the most widely used bioceramic material for bone grafting in humans [16] due to its chemical composition and crystalline structure that closely resemble those of natural bone [17,18]. HAP and other calcium-based ceramic materials are considered bioactive as they support bone growth [19,20,21]. Their bioactivity is linked to osteoconductive properties that facilitate the attachment and migration of osteoblasts to the material surface [22,23]. In addition, HAP is known to form direct bonds with bone [24].

Despite numerous advantages, the long-term regenerative effects of a bone substitute composed of HAP and tricalcium phosphate (TCP) have not been thoroughly analyzed in a clinical setting. Thus, this study aims to assess whether HAP-TCP has a significant influence on alveolar bone regeneration during maxillary sinus augmentation. The clinical analysis was conducted using imaging, functional assessments, and clinical observations to evaluate the quality of subantral augmentation using the Nano Graft composite, and to predict implantation outcomes in the augmented regions.

## 2. Materials and Methods

### 2.1. Study Design

This cross-sectional study included 22 patients, aged 39 to 75, undergoing rehabilitation for secondary partial edentulousness through dental implantation (Nano Prime PEO, Debica, Poland) and subantral augmentation in the maxillary sinus region using HAP-TCP Nano Graft composite (Nano Prime, Debica, Poland) [25]. A total of 52 augmentation sites and 67 native bone sites were analyzed. For comparison, both augmented and native bone were harvested from the same implant site. This approach controls anatomical variations across different patients and sinus regions. Exclusion criteria included any generalized systemic diseases or systemic antibiotic treatment within 3 months prior to the study. Informed consent was obtained from all participants. Ethical approval was granted by the Research Ethics Committee of Zaporizhzhia State Medical and Pharmaceutical University (Bioethics Commission ZDMFU, protocol No. 5, Approval Date: 10 November 2022). All sinus augmentation and implant placement procedures were performed by a single experienced surgeon following a uniform surgical protocol. This was an observational cohort study and was not registered in a clinical trials registry, as registration is not required for non-interventional designs under local regulations.

### 2.2. Assessment of Regenerated Bone Quality at Implant Placement

The quality of the augmented site was evaluated at the time of implant placement using multiple metrics: (1) bone type according to the Misch classification, (2) intraoperative bleeding response, (3) radiographic bone density measured in Hounsfield Units (HU), and (4) insertion torque (measured in N·cm). These indicators were systematically compared to the corresponding values observed in the patient’s native bone at the same or adjacent anatomical sites, enabling an assessment of the relative quality and clinical suitability of the augmented bone for implant support. Additionally, bone biopsies were obtained from a standardized depth within the augmentation area for subsequent morphological analysis. All intraoperative assessments were performed by the operating surgeon. However, no independent observers were involved in these assessments, which may be a source of observer bias, as acknowledged in the discussion chapter.

#### 2.2.1. Determination of Bone Type by Misch

Bone quality was assessed according to the Misch bone classification system, a widely accepted framework for evaluating macroscopic bone density in the context of dental implantology [26,27,28].

The Misch classification delineates bone into four principal types based on macroscopic density and structure:

- D1: Dense cortical bone;
- D2: Thick, porous cortical bone with coarse trabecular bone;
- D3: Thin cortical bone with fine trabecular structure;
- D4: Predominantly thin trabecular bone with minimal cortical component;
- D5: (occasionally referenced): Immature, poorly mineralized trabecular bone, typically observed in early healing phases.

#### 2.2.2. Bone Density

The qualitative characteristics of both native and augmented bone tissues were evaluated using computed tomography (CT) and quantified based on the Hounsfield Unit (HU) scale. All CBCT scans were performed in the same radiology unit using NewTom Go 70-BE and NewTom GiANO HR systems (Cefla s.s., Imola, Italy) with standardized acquisition parameters (fixed voltage, current, field of view, and voxel size) according to the manufacturer’s recommendations. The devices undergo routine calibration and quality-assurance procedures as part of clinical practice, ensuring stability of gray-value output over time. For each patient, augmented and native bone regions were assessed within the same CBCT examination, thereby minimizing variability related to scanner settings, beam hardening, and reconstruction algorithms. In this context, HU values were used as semi-quantitative indicators of relative radiodensity within a single scan rather than as absolute measures of bone mineral density.

CT image analysis software was used to calculate the average radiodensity within predefined regions of interest (ROI). For each implant site, a circular ROI of fixed diameter was placed in the cancellous compartment of the augmented area, centered at the planned implant bed and positioned approximately 7–10 mm apical to the alveolar crest, while carefully excluding the cortical borders of the sinus and any visible artifacts from metallic restorations or graft granules. An identically sized ROI was then mirrored and positioned in the adjacent native bone on the same slice at a corresponding vertical level. When necessary, three consecutive slices encompassing the implant site were evaluated and the mean HU value was calculated. This standardized ROI protocol was applied identically to both augmented and native bone regions to reduce variability arising from ROI size, location, and sinus anatomy. The HU values were employed to classify bone quality according to an extended Misch-based system [29,30]:

- D1: >1250 HU (very dense cortical bone);
- D2: 850–1250 HU (dense to porous cortical bone with coarse trabecular structure);
- D3: 350–850 HU (thin cortical bone with denser trabecular structure);
- D4: 150–350 HU (primarily trabecular bone with minimal cortical component);
- D5: <150 HU (very soft or immature bone, often observed in newly grafted sites).

#### 2.2.3. Homogeneity of Bone Structure and Vascularization

The uniformity of the bone structure was assessed after forming the implant site. Additionally, rinsing the site with an antibiotic confirmed that no disorganized augmentation fragments were present. Bone structure homogeneity during the preparation of the implant site was determined manually at each drilling stage and assessed through torque measurements of the rotating instrument.

Bleeding from both the augmentation and native bone tissues was assessed during the final drilling process as suggested in the study by Ghadimi [31], and Halme [32]. Bleeding was classified into two grades:

- Grade I: Slight bleeding (single-point bleeding);
- Grade II: Intense bleeding (intense bleeding immediately following the drill’s passage through the augmentation site).

This grading of bleeding represented a purely qualitative, intraoperative assessment of local perfusion at the osteotomy site. It was used as a simple clinical descriptor to compare augmented and native bone within the same patient and under the same drilling protocol. Importantly, this visual grading does not provide a validated quantitative measure of angiogenesis or vessel density and is therefore interpreted only as an adjunctive indicator rather than a direct surrogate for vascularization.

#### 2.2.4. Insertion Torque

The primary stability of dental implants was evaluated by measuring the insertion torque at the time of placement using a calibrated torque-controlled handpiece. Insertion torque reflects the degree of bone compression and is a key parameter for establishing immediate mechanical contact between the implant surface and the surrounding bone tissue. This mechanical interaction is critical for primary stability and subsequent osseointegration. The primary stability of the implant was measured by the torque measured during placement (measured in N·cm) as documented in studies by Yamaguchi [33] and do Vale Souza [34]. A torque threshold of ≥35 N·cm was considered indicative of adequate primary stability, consistent with established clinical guidelines. Torque values below this threshold were categorized as insufficient for achieving reliable primary stability and documented accordingly for comparative analysis.

To minimize the influence of implant- and technique-related variables on insertion torque, all implants were selected from the same implant system sharing the same macrodesign and thread geometry. Implant sites were prepared using the standard manufacturer’s sequential drilling protocol with pilot, intermediate, and final drills of increasing diameter under copious irrigation. For each planned implant diameter, the final osteotomy was intentionally kept slightly smaller than the implant diameter (controlled under-preparation) in both augmented and native bone to achieve press-fit primary stability. Osteotomy depth and implant positioning were planned to obtain primary cortical contact at the crestal alveolar ridge, without intentional bicortical anchorage against the sinus floor. All surgeries were performed by the same experienced operator using this uniform protocol, so that differences in insertion torque primarily reflected local bone conditions rather than variations in implant design or drilling technique.

### 2.3. Statistical Analysis

Statistical analysis of the post-augmentation metrics for compliance with the normal distribution was conducted using the Shapiro–Wilk test. Within the augmentation group, and torque differences based on bone type were assessed using the Mann–Whitney test. Within the native bone group, torque differences based on bone type were assessed using the Kruskal–Wallis test. Data exhibiting Gaussian distribution were presented as mean ± standard deviation (SD), whereas non-Gaussian data were expressed as median with interquartile range (IQR). The Mann–Whitney U test was used to analyze differences between groups with non-Gaussian distribution. Statistical significance was considered at *p* < 0.05.

The reporting of this observational study is in accordance with the STROBE statement checklist.

## 3. Results

### 3.1. Time Range Analysis

**Figure 1:**
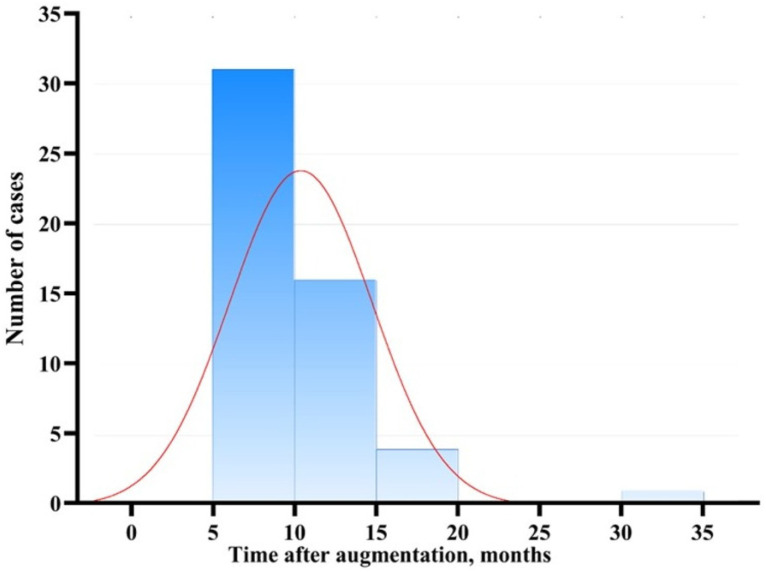
Duration of follow-up for evaluation of newly formed bone tissue. Box-and-whisker plot of the time interval between subantral augmentation with HAP-TCP and implant placement in the augmented sites.

The newly formed bone tissue was evaluated over a period ranging from 6.5 to 34 months following implant placement (Figure 1). This variability primarily stemmed from individual patient-related factors. Notably, the duration of exposure—within the 6.5 to 34-month interval—did not influence the primary criteria used for the qualitative assessment of regenerated bone tissue.

The distribution of the indicator deviated significantly from normality, as confirmed by the Shapiro–Wilk test (*p* < 0.001). The median implantation period was 9.5 months, with an interquartile range (Q25; Q75) of 8.00 to 11.75 months.

No significant correlation was found between the time elapsed after augmentation and bone density (Spearman’s ρ = 0.084; *p* > 0.05). Similarly, there was no significant correlation between the augmentation period and bone type (ρ = –0.109; *p* > 0.05), or with insertion torque (ρ = 0.154; *p* > 0.05).

### 3.2. Determination of Bone Type by Misch

**Figure 2:**
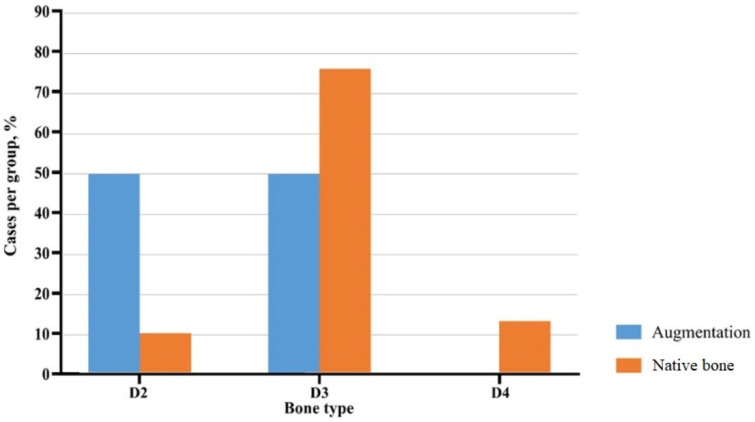
Distribution of bone tissue according to Misch bone types. Proportion of implant sites classified as D2, D3, or D4 bone in the HAP-TCP-augmented and native bone groups.

The results indicate a heterogeneous structure of the maxillary alveolar ridge, characterized by the distribution of Misch bone types D2, D3, and D4. In the augmentation group, bone type D2 was present in 10% of cases, with bone type D3 being predominant at 76%. Notably, bone type D4 was absent in the augmentation group. In contrast, the native bone tissue exhibited 11% of bone type D2, 80% of bone type D3, and 9% of bone type D4. The absence of Misch bone type D4 in the augmentation group compared to native bone tissue underscores the variability in bone composition between these two groups (Figure 2).

These results highlight the differences in bone composition between the augmentation and native bone groups (Table 1). The augmentation group shows a higher prevalence of bone type D2 (50.0%) compared to the native bone group (10.4%), with a significant *p*-value of <0.0001. Bone type D3 was equally prevalent in the augmentation group but was more common in the native bone group (*p* = 0.0030). Bone type D4 was absent in the augmentation group but present in 13.4% of the native bone group, with a *p*-value of 0.0061.

### 3.3. Bone Density

The analysis of the density characteristics of the augmentation revealed an average radiodensity of 1132.6 ± 334.9 HU, which was 45.9% higher than that of the patient’s adjacent native bone in the same posterior maxillary segment (519.3 ± 395.0 HU; *p* < 0.0001). These values reflect relative intra-scan differences between augmented and native bone regions obtained under identical CBCT acquisition conditions.

### 3.4. Homogeneity of the Bone Structure and Determination of Degree of Vascularization

Along with density characteristics, homogeneity of bone structure and the degree of vascularization are crucial indicators of bone tissue quality. These factors play a pivotal role in the successful integration of bone tissue with the implanted structure [35,36].

Analysis of bone structure homogeneity indicated that 69.2% of the augmentation group displayed a homogeneous structure compared to 3.0% of the native bone group. The heterogeneity of the native bone structure (97.0%) aligns with established anatomical data regarding the maxilla (Table 2).

Profuse bleeding in the augmentation zone was detected in 67.3% of instances, while slight bleeding was noted in 32.7% of observations (Table 2).

The augmentation zone utilizing the synthetic bone composite contained a higher proportion of sites with intense intraoperative bleeding than the native bone (67.3% vs. 47.8%; Table 2). Under a uniform drilling protocol, this finding suggests a more richly perfused surgical bed in the regenerated area at the time of implant placement. However, because bleeding intensity was assessed visually and in a binary fashion, these data should be interpreted as a qualitative indication of intraoperative perfusion, rather than as a direct or quantitative measure of angiogenesis or microvascular network formation.

### 3.5. Insertion Torque

Analysis of torque values in the regions of newly formed (augmented) bone yielded an average torque value of 35 N·cm (interquartile range [Q25–Q75]: 35–40) across 52 observations, which was significantly greater compared to that in areas of the patient’s native bone directly adjacent to the augmentation zone at 25 N·cm (interquartile range: 25–30) (*p* < 0.0001).

**Figure 3:**
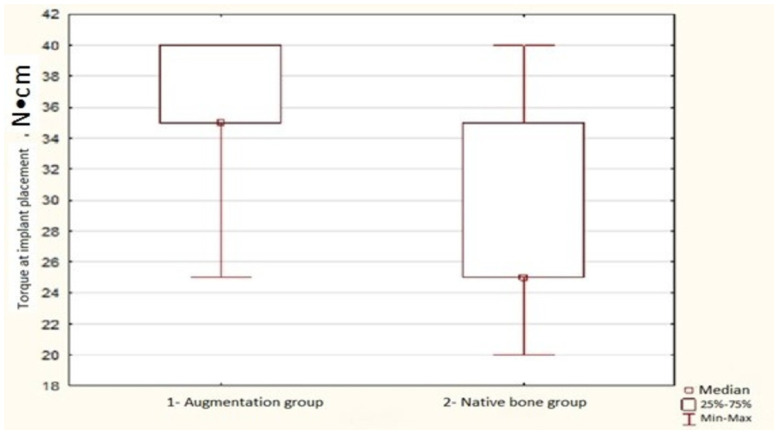
Comparison of insertion torque between augmented and native bone. Box-and-whisker plot of insertion torque values for implants placed in HAP-TCP-augmented bone and in adjacent native bone in the posterior maxilla.

Comparative analysis of torque values during implant placement was performed across comparative groups in the areas of augmentation and native bone. Additionally, torque values were evaluated within each group to determine the correlation between bone density (in HU) and torque values (in N·cm). The results of this analysis are illustrated in Figure 3.

**Figure 4:**
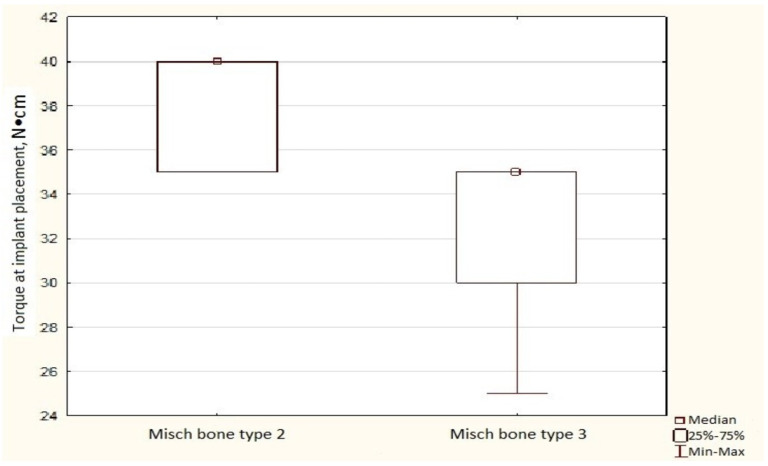
Insertion torque according to Misch bone type in augmented bone. Box-and-whisker plot of insertion torque values in HAP-TCP-augmented bone, stratified by Misch bone type.

During implant placement, we also found a correlation between the structural characteristics of the Misch bone and torque values. In the augmentation group, higher bone type corresponded to lower torque values (Kendall’s correlation coefficient R = −0.686 *p* < 0.05). Specifically, in bone type D2, the median torque was 33.3%, which was higher than that in bone type D3 (Figure 4).

**Figure 5:**
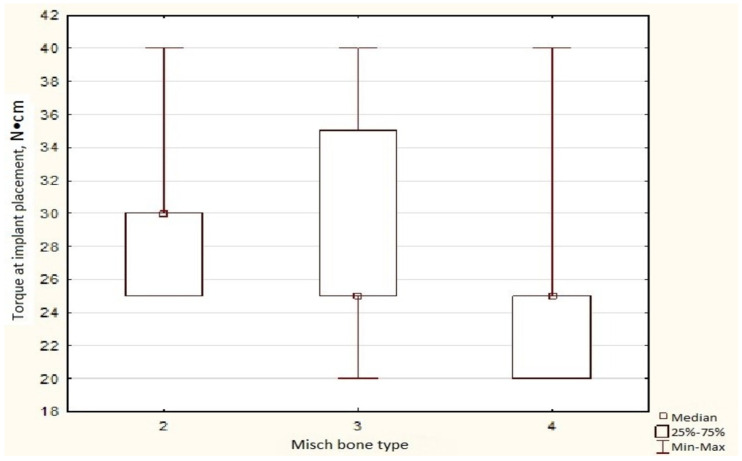
Insertion torque according to Misch bone type in native bone. Box-and-whisker plot of insertion torque values in native bone, stratified by Misch bone type.

Moreover, the native bone group also exhibits the same inverse correlation, with higher bone type corresponding to a lower torque value (Kendall’s correlation coefficient R = −0.295 *p* < 0.05). In bone type D2, the median torque is 20.0% higher than that in bone types D3 and D4 (Figure 5).

## 4. Discussion

The use of synthetic bone composites, such as HAP-TCP, has demonstrated effectiveness in maintaining vertical bone growth, which is essential for successful implant placement following sinus lift procedures [37,38]. The results of this study support the use of HAP-TCP, showing that bone quality remained stable whether implants were placed at 6.5 months or up to 34 months post-augmentation. Specifically, factors such as bone type, bone homogeneity, bleeding, bone density, and torque during implant placement all demonstrated no statistically significant differences associated with the duration of augmentation (*p* > 0.05 for all comparisons). This observation suggests that the integrity and quality of bone tissue formed through HAP-TCP remain stable over time within the studied exposure range. Furthermore, the effectiveness of the synthetic bone composite (HAP-TCP) in maintaining vertical bone growth has been demonstrated, allowing for implant placement as early as 6 months after sinus lift [23].

A fundamental consideration in implantology is bone quality, as areas with suboptimal bone quality tend to have higher rates of implant failure [39]. The distinction between cortical and cancellous bone is fundamental to understanding implant stability. Cortical bone is essential for initial implant stability, whereas cancellous bone is crucial for long-term recovery and osseointegration [40]. The majority of an implant’s surface is in contact with cancellous bone, underscoring the importance of this tissue type in successful implant placement [41]. Furthermore, a combination of high bone density and adequate vascularization within the augmentation zone indicates well-organized bone tissue, conducive to the implant procedure. Conversely, areas exhibiting irregularities or “dips” in bone structure indicate less organized tissue, potentially complicating the implant placement process and diminishing the predictability of osseointegration.

Our results demonstrate that subantral augmentation with a synthetic bone composite comprising HAP-TCP is associated with a 45.9% higher radiodensity in HU compared to adjacent native bone in the same posterior maxillary segment. This radiodensity difference, together with the higher prevalence of Misch D2/D3 bone types, greater structural homogeneity, more intense intraoperative bleeding, and higher insertion torque values, suggests that the regenerated tissue provides a functionally favorable environment for implant placement at the time of surgery. Higher radiodensity is likely attributable to the high content of HAP within the augmentation zone, which promotes formation of denser bone tissue, even though trabecular organization may remain heterogeneous in some areas. HAP’s recognized biocompatibility and osteoconductive properties facilitate increases in bone density and support tissue regeneration [42,43,44]. However, this 45.9% higher HU value should be interpreted as a semi-quantitative indicator of local X-ray attenuation during implant placement, rather than as a direct percentage of “improvement in bone quality” or a guarantee of superior long-term clinical outcomes.

Several systematic reviews and clinical studies on maxillary sinus floor augmentation using autogenous bone, deproteinized bovine bone mineral (DBBM), β-tricalcium phosphate (β-TCP), and biphasic calcium phosphate grafts have reported high implant survival and broadly comparable histomorphometric outcomes among materials [9,10,11]. Raghoebar et al. reported an annual implant loss of approximately 0.4% and no clear differences in survival between autogenous and substitute grafts in the posterior maxilla [45] while Starch-Jensen et al. showed that autogenous bone tends to yield slightly higher vital bone fractions than allogeneic, xenogeneic, or synthetic substitutes, but with only small clinical differences in implant stability and survival [46]. Prospective and randomized studies on maxillary sinus augmentation with β-TCP and biphasic HA/β-TCP have demonstrated new bone formation in the range of roughly 30–50% after 6–12 months and implant survival rates of ≥95–97% [25,37]. HAP–TCP outcomes appeared broadly comparable to autogenous bone and xenografts, specifically regarding the D2–D3 radiodensity range and mean insertion torque of 35 N·cm. This indicates that the material performs effectively within the standard range, without demonstrating clear superiority [23,37].

Bone density, in conjunction with structural homogeneity and vascularization, plays a pivotal role in the success of bone augmentation [47]. Dense bone, particularly within the 7–10 mm region from the ridge margin, is critical for successful implant placement. This study revealed that the distribution of Misch type D2 and type D3 bone within the augmentation group is indicative of stable bone remodeling processes that favor the predominance of denser bone types conducive to implantation. The absence of Misch type D4 bone, which typically displays less predictability and reliability for implant placement, further bolsters the favorable outcomes observed in the augmentation group. Notably, the presence of dense cortical bone and the capability of trabecular bone to fulfill the implant’s torque requirements significantly contribute to the long-term success of the procedure. Previous research has highlighted that cortical bone significantly contributes to the insertion torque value, which is essential for primary implant stability [48].

The torque required for implant placement is another clinically relevant surrogate of primary stability [49,50]. However, insertion torque is not determined by bone alone. It is also influenced by implant macrodesign, thread geometry, drill protocol, and the depth of cortical engagement. In the present study, these technical parameters were standardized, as the same implant system, drilling sequence with controlled under-preparation, and crestal cortical contact were used in both augmented and native bone. Under these conditions, the higher torque values observed in the regenerated area indicate improved primary stability under a uniform protocol, but they should not be interpreted as direct proof of intrinsically “superior” bone quality. Instead, insertion torque should be considered together with radiodensity, structural homogeneity, vascularization, and histological findings when evaluating the performance of the augmented tissue [51,52,53].

In addition to bone density and homogeneity, vascularization plays an important role in osseointegration and long-term implant success [54]. In the present study, however, vascularization was not quantified directly. Instead, we used intraoperative bleeding during osteotomy preparation as a simple, qualitative descriptor of local perfusion [45,46]. The greater frequency of intense bleeding observed in the augmented region indicates a more perfused surgical bed under a standardized drilling protocol, but this observation cannot be equated with increased angiogenesis or microvascular density. Thus, the bleeding data should be regarded as supportive clinical information and interpreted cautiously, with definitive conclusions about vascularization reserved for future studies incorporating dedicated histological or micro-CT analyses of the vascular network [55].

Although the present follow-up period of 6.5–34 months is limited to early and mid-term healing, available long-term data on sinus augmentation with β-TCP and biphasic calcium phosphate (BCP) suggest that these synthetic grafts can support high implant survival (≈95–97%) and relatively stable marginal bone levels over ≥5–10 years. In a 10-year series of maxillary sinus floor augmentation using pure β-TCP, implant survival was 97.2% with mean marginal bone loss of about 2 mm, and most vertical height reduction occurred during the first 1–2 years, with only minimal changes thereafter. Systematic reviews of long-term sinus floor augmentation likewise report high implant survival and limited peri-implant bone loss beyond 5 years, while emphasizing that continued remodeling and some reduction in graft height can occur over time and that robust long-term randomized data are still scarce. Histological case reports of HA/β-TCP scaffolds retrieved after 7 years show mature lamellar bone intimately integrated with residual BCP particles, consistent with slow resorption of the TCP phase and ongoing remodeling rather than complete graft substitution. On this basis, it is plausible that the HAP–TCP composite used in the present study will continue to remodel beyond 34 months, with progressive replacement of the more soluble TCP fraction by vital bone while the HA component contributes to volumetric stability. However, our data do not allow conclusions on clinical performance beyond the current observation period.

This study had certain limitations. First, it involved a relatively small sample size of 22 patients, limiting the generalizability of the results. Future studies should include a larger cohort of participants to enhance statistical robustness and validate the findings across diverse patient populations. Second, this study assessed outcomes within a range of 6.5 to 34 months post-augmentation, which may not be sufficient to ascertain the long-term stability and efficacy of HAP-TCP in bone regeneration and implant success. Subsequent studies should conduct extended follow-up evaluations beyond 36 months. Consequently, the higher radiodensity and insertion torque observed in the augmented region should be understood reflecting early intraoperative conditions and improved primary stability under a standardized protocol, rather than as definitive evidence of enhanced long-term implant survival or overall treatment success. Third, bone quality was assessed by comparing augmented areas to adjacent native bone within the same patient, which may have introduced inherent bias due to local physiological factors affecting healing and bone quality. Utilizing a randomized, controlled design with a parallel-group approach could provide independent comparisons, further mitigating the biases of intra-patient comparisons. Insertion torque is likewise affected by implant design and surgical technique in addition to bone properties. Although we reduced this bias by using one implant system and a standardized drilling and under-preparation protocol, insertion torque in this study should still be regarded as an indirect surrogate of local bone conditions rather than an isolated measure of intrinsic bone quality. Fourth, CBCT-derived HU values are inherently scanner-dependent and influenced by beam hardening, sinus anatomy, and ROI definition. Consequently, HU values in this study should be interpreted as semi-quantitative indicators of X-ray attenuation rather than absolute measures of bone mineralization. To mitigate this limitation, all comparisons between augmented and native bone were made within the same scan using a standardized ROI protocol. Furthermore, radiodensity data were interpreted in conjunction with Misch bone type, intraoperative bleeding, homogeneity of the bone structure, insertion torque, and histological findings. In addition, vascularization was evaluated only qualitatively by grading intraoperative bleeding during drilling, which is not a validated surrogate measure of angiogenesis or vessel density. As a result, our conclusions regarding vascularization should be considered exploratory, and future investigations incorporating dedicated histological or micro-CT assessment of the vascular network are needed to more accurately characterize neovascularization in the augmented sinus. Finally, although the surgical procedures and implant system were standardized, residual variability in surgical technique and patient-specific healing capacity cannot be excluded, and the single-arm, intra-patient comparative design limits the ability to attribute the observed differences in radiodensity and insertion torque solely to the HAP-TCP composite. Accordingly, these percentage differences should be viewed as indicative of correlations observed under a uniform protocol rather than as conclusive evidence of intrinsic superiority of the regenerated bone.

In conclusion, within the limits of this cross-sectional observational study, the application of HAP-TCP for subantral bone augmentation was associated with favorable parameters of bone quality, radiodensity, and intraoperative perfusion at implant placement. The synthetic bone composite was associated with a 45.9% higher radiodensity in the augmented region compared to adjacent native bone and a 19.5% increase in intraoperative indicators of perfusion, together with greater structural homogeneity and higher insertion torque values under a standardized surgical and implant protocol. The 71.4% higher torque observed in the augmentation zone therefore reflects greater primary stability under these conditions, rather than definitive evidence of intrinsically superior mechanical properties of the regenerated bone. These findings underscore the potential of HAP-TCP as a reliable material for sinus augmentation while emphasizing that the observed percentage differences represent associations rather than proof of a causal, material-specific effect.

## Figures and Tables

**Table 1:** Distribution of Misch bone types at implant sites. Number and percentage of implant sites classified as D2, D3, or D4 in the HAP-TCP-augmented and native bone groups.

Bone Type According to K. Misch	Augmentation Group, (*n* = 52)	Native Bone Group, (*n* = 67)	*p*-Value *
D2	26/50.0	7/10.4	<0.0001
D3	26/50.0	51/76.2	0.0030
D4	0/0.0	9/13.4	0.0061

Data are expressed as absolute numbers and percentages (n/%). * *p*-value < 0.05 is considered to be statistically significant.

**Table 2:** Bone structure homogeneity and intraoperative bleeding in augmented and native bone. Comparison of homogeneous vs. non-homogeneous bone structure and intense vs. slight bleeding during drilling in HAP-TCP-augmented and native bone regions.

Indicator	Augmentation Group (*n* = 52)	Native Bone Group (*n* = 67)	*p*-Value *
Homogeneous consistency, n/%	36/69.2	2/3.0	<0.0001
Nonhomogeneous consistency, n/%	16/30.8	65/97.0	<0.0001
Intense bleeding, n/%	35/67.3	32/47.8	0.0334
Slightly bleeding, n/%	17/32.7	35/52.2	0.0334

* *p* < 0.05 is considered to be statistically significant.

## Data Availability

The original contributions presented in this study are included in the article. Further inquiries can be directed to the corresponding authors.
